# Antifungal Activity of Thirty Essential Oils to Control Pathogenic Fungi of Postharvest Decay

**DOI:** 10.3390/antibiotics13010028

**Published:** 2023-12-28

**Authors:** Mohamed Bechir Allagui, Marwa Moumni, Gianfranco Romanazzi

**Affiliations:** 1 Laboratory of Plant Protection, National Institute for Agronomic Research of Tunisia (INRAT), University of Carthage, Rue Hedi Karray, Ariana 2080, Tunisia; 2 Department of Agricultural, Food and Environmental Sciences, Marche Polytechnic University, Via Brecce Bianche, 60131 Ancona, Italy; m.moumni@staff.univpm.it (M.M.); g.romanazzi@univpm.it (G.R.)

**Keywords:** blue mold, essential oils, fruits, gray mold, shelf life

## Abstract

Essential oils (EOs) extracted from aromatic or medicinal plants are biodegradable, safe, and regarded as alternatives to chemical pesticides to reduce fungal species attacking different crops. In this study, thirty EOs at 0.5 mg/mL were evaluated for in vitro growth inhibition of the main postharvest fungi, which are *Alternaria alternata*, *Botrytis cinerea*, and *Penicillium italicum*. *Cinnamomum verrum* EO completely inhibited the mycelial growth of *A. alternata* and *B. cinerea*, and *Syzygium aromaticum* EO completely inhibited the mycelia of *A. alternata*. *B. cinerea* mycelial growth was completely inhibited by *Gautheria fragrantissima*, *Cymbopogon nardus*, *Pelargonium asperum*, and *Cupressus sempervirens* EOs. *G. fragrantissima* EO inhibited the mycelia growth of *P. italicum* by 98%. Overall, *B. cinerea* displayed the highest sensitivity to EOs than *P. italicum* and *A. alternata*. *G. fragrantissima*, *C. sempervirens*, *C. nardus*, *P. asperum*, *Mentha piperita*, *Foeniculum vulgare*, *C. verrum*, and *S. aromaticum* EOs showed the highest inhibition for these three pathogens. Minimum inhibitory concentrations were lower for *C. verrum* and *S. aromaticum* EOs, ranging between 0.31 and 0.45 mg/mL and 0.37 to 0.57 mg/mL, respectively, against the three pathogens. The tested EOs inhibited the in vitro growth of three of the main postharvest fungal pathogens. Further studies are needed to confirm these activities in vivo.

## 1. Introduction

Food loss and waste are issues of importance to global food security, and according to the Food and Agriculture Organization of the United Nations, 45% of all fruits and vegetables are lost or wasted every year [1]. This waste occurs along the entire food chain (from field to consumer) and needs to be analyzed and monitored due to its impact on the development of the food sector. Contamination of fruit and vegetables by pathogenic microorganisms is a major factor in reducing yields and market quality. The use of fungicides is a common practice as a postharvest treatment to control fruit decay. In recent years, it has been necessary to achieve the United Nations’ Sustainable Development Goals (SDGs) and the Farm to Fork Strategy of the European Green. In addition, fresh fruit loss, waste prevention, and management are included among the 17 SDGs (targets 12.2 and 12.3) within Agenda 2030 for Sustainable Development. Also, the Commission under the Farm to Fork Strategy of the European Green Deal aims to reduce the use of dangerous compounds in agriculture and achieve at least 25% of the EU’s agricultural land under organic farming by 2030. Nowadays, environment-friendly alternatives, such as essential oils, are being developed and tested for antifungal activities against postharvest pathogens. Essential oils (EOs) are a rich source of broad-spectrum antifungal plant-derived metabolites that inhibit both fungal growth and their production of toxic metabolites [2,3,4].

EOs are extracted from different plant species to evaluate the bioactive compounds able to extend the shelf life of packed foods. Some of these EOs that displayed effectiveness in reducing fungal decay are tea seed [5], camellia [6], oregano [7], cinnamon [8], lemongrass [9], sunflower seed [10], *Citrus sinensis* [11], *Ziziphora persica* [12], clove [13], and fennel [14]. As a result, the production and consumption of essential oils have expanded over the world in recent years [15]. The total EO content of plants is generally low and rarely exceeds 1%, with the exception of some cases, for example, clove (*Syzygium aromaticum*) and nutmeg (*Myristica fragrans*), which could reach 10% [16].

There are limitations to incorporating these EOs, such as their low solubility in water, high volatility, and possible toxicity at high concentrations [17]. Despite these drawbacks, they present numerous positive aspects in food packaging, such as the promotion of food shelf life, inhibition of microbiological deterioration by fungi and bacteria, and delay in fruit ripening [17]. Recently, there has been a great interest in using EOs as possible natural substitutes for conventional synthetic fungicides [18,19]. Plants rich in terpenes, alkaloids, or phenolic compounds with low or no residual effect could act as bioinsecticides, biofungicides, and bioherbicides to replace the classical chemicals and reduce the impact on the environment [20,21].

EO components could act as antifungal agents due to their accumulation in the lipophilic hydrocarbon molecules of the cell lipid bilayer; such action also allows the easier transfer of other EO constituents to the inner part of the cell. Water solubility and lipophilic properties of the EOs may explain their difference in activities [22].

In this study, thirty EOs were analyzed in vitro regarding their efficacy toward three fungal species responsible for different rots on harvested fruits and during cold storage. These fungal species were classified according to their general reaction against thirty EOs extracted from varied plant species before determining the MIC required.

## 2. Results

### 2.1. Response of Alternaria alternata towards 30 EOs

**Figure 1:**
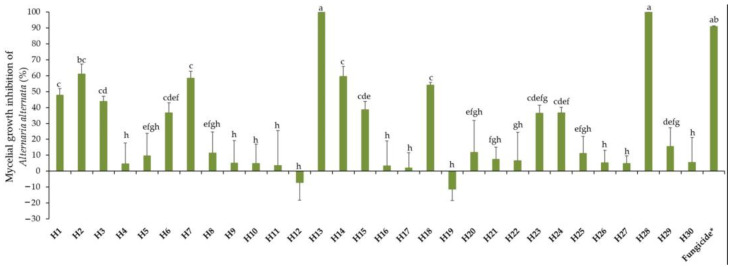
Mycelial growth inhibition of *A. alternata* by thirty essential oils used at 0.5 mg/mL. H1, *C. martinii*; H2, *F. vulgare*; H3, *L. nobilis*; H4, *M. alternifolia*; H5, *S. sclarea*; H6, *C. odoranta*; H7, *P. cablin*; H8, *M. quinquenervia*; H9, *B. carterii*; H10, *L. angustifolia*; H11, *C. sinensis*; H12, *C. paradisii*; H13, *S. aromaticum*; H14, *M. piperita*; H15, *G. fragrantissima*; H16, *A. dracunculus*; H17, *C. limon*; H18, *D. carota*; H19, *Z. officinale*; H20, *P. crispum*; H21, *C. reticulata*; H22, *C. aurantium bergamia*; H23, *C. nardus*; H24, *P. asperum*; H25, *J. communis*; H26, *C. nobile*; H27, C. atlantica; H28, C. verrum; H29, C. sempervirens; H30, *O. basilicum*, after 21 days of incubation at 10 °C in the dark. * 25 g/L difenoconazole + 25 g/L fludioxonil. Data are expressed as means ± Standard Error (SE, n = 3). Data with different letters are significantly different (*p* ≤ 0.05, Fisher’s LSD tests).

**Figure 2:**
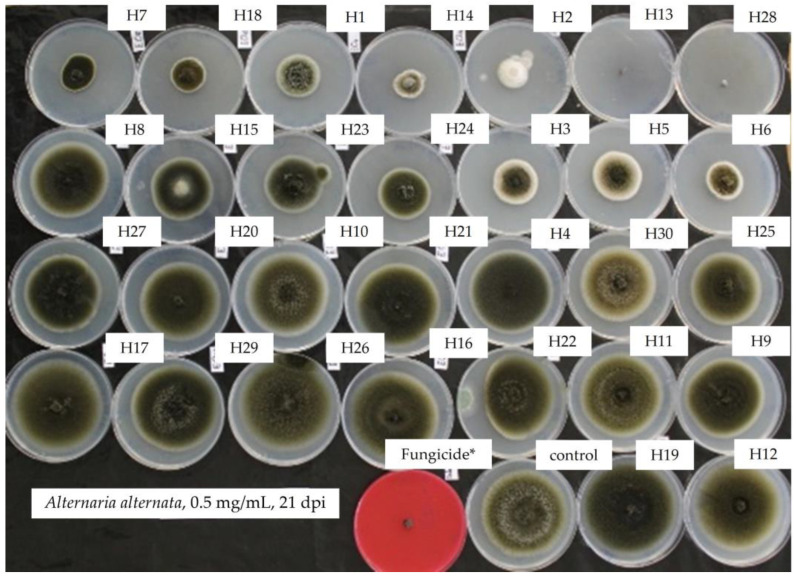
Mycelial growth inhibition of *A. alternata* in PDA amended with the essential oils at 0.5 mg/mL showing total inhibition of mycelia (upper) and progressively normal growth (bottom) (red plate amended with difenoconazole + fludioxonil showing total inhibition). H1, *C. martinii*; H2, *F. vulgare*; H3, *L. nobilis*; H4, *M. alternifolia*; H5, *S. sclarea*; H6, *C. odoranta*; H7, *P. cablin*; H8, *M. quinquenervia*; H9, *B. carterii*; H10, *L. angustifolia*; H11, *C. sinensis*; H12, *C. paradisii*; H13, *S. aromaticum*; H14, *M. piperita*; H15, *G. fragrantissima*; H16, *A. dracunculus*; H17, *C. limon*; H18, *D. carota*; H19, *Z. officinale*; H20, *P. crispum*; H21, *C. reticulata*; H22, *C. aurantium bergamia*; H23, *C. nardus*; H24, *P. asperum*; H25, *J. communis*; H26, *C. nobile*; H27, *C. atlantica*; H28, *C. verrum*; H29, *C. sempervirens*; H30, *O. basilicum*, after 21 days of incubation at 10 °C in the dark. * 25 g/L difenoconazole + 25 g/L fludioxonil.

Mycelial growth of *A. alternata* in PDA was determined 21 days post incubation at 10 °C in the dark. The activity of EOs towards *A. alternata* varied considerably, from zero to high efficacy, as expressed in Figure 1, showing a total lack of efficiency from −11% (H19, *Z. officinale*) to a total efficacy of 100% (H13, *S. aromaticum* and H28, *C. verrum*). Negative values for such an EO indicate that it is without any antifungal activity; on the contrary, it stimulates fungal growth better than the control, thus playing the role of a nutrient favorable to fungal growth. Intermediate reactions were expressed by different EOs. Mycelial growth inhibition by respective EOs can be visualized in Figure 2.

### 2.2. Response of Botrytis cinerea towards 30 EOs

**Figure 3:**
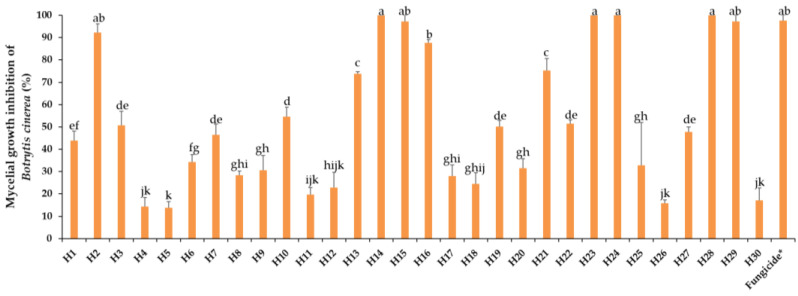
Mycelial growth inhibition of *B. cinerea* by the thirty essential oils tested at 0.5 mg/mL. H1, *C. martinii*; H2, *F. vulgare*; H3, *L. nobilis*; H4, *M. alternifolia*; H5, *S. sclarea*; H6, *C. odoranta*; H7, *P. cablin*; H8, *M. quinquenervia*; H9, *B. carterii*; H10, *L. angustifolia*; H11, *C. sinensis*; H12, *C. paradisii*; H13, *S. aromaticum*; H14, *M. piperita*; H15, *G. fragrantissima*; H16, *A. dracunculus*; H17, *C. limon*; H18, *D. carota*; H19, *Z. officinale*; H20, *P. crispum*; H21, *C. reticulata*; H22, *C. aurantium bergamia*; H23, *C. nardus*; H24, *P. asperum*; H25, *J. communis*; H26, *C. nobile*; H27, C. atlantica; H28, C. verrum; H29, C. sempervirens; H30, *O. basilicum*, after 8 days of incubation at 10 °C in the dark. * 25 g/L difenoconazole + 25 g/L fludioxonil. Data are expressed as means ± SE (n = 3). Data with different letters are significantly different (*p* ≤ 0.05, Fisher’s LSD tests).

**Figure 4:**
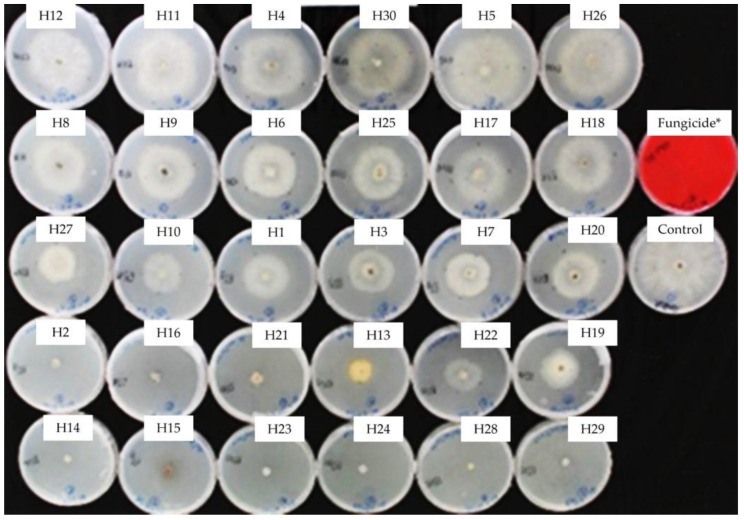
Mycelial growth inhibition of *B. cinerea* in PDA amended with the essential oils used at 0.5 mg/mL. H1, *C. martinii*; H2, *F. vulgare*; H3, *L. nobilis*; H4, *M. alternifolia*; H5, *S. sclarea*; H6, *C. odoranta*; H7, *P. cablin*; H8, *M. quinquenervia*; H9, *B. carterii*; H10, *L. angustifolia*; H11, *C. sinensis*; H12, *C. paradisii*; H13, *S. aromaticum*; H14, *M. piperita*; H15, *G. fragrantissima*; H16, *A. dracunculus*; H17, *C. limon*; H18, *D. carota*; H19, *Z. officinale*; H20, *P. crispum*; H21, *C. reticulata*; H22, *C. aurantium bergamia*; H23, *C. nardus*; H24, *P. asperum*; H25, *J. communis*; H26, *C. nobile*; H27, C. atlantica; H28, C. verrum; H29, C. sempervirens; H30, *O. basilicum*, after 8 days of incubation at 10 °C in the dark. * 25 g/L difenoconazole + 25 g/L fludioxonil.

Mycelial growth of *B. cinerea* in PDA was rated after 8 days of incubation at 10 °C in the dark. The activity of the EOs towards *B. cinerea* varied greatly from highly to weakly effective, as represented in Figure 3, showing a progressive efficacy from 22% (H5) to 100% (H28, H23, H29, H24, and H15). Nine (H2, H14, H15, H16, H23, H24, H28, and H29) EOs were effective in controlling this fungus, and there was no significant difference between them and the fungicide. Intermediate reactions were expressed by different EOs. Mycelial growth inhibition by respective EOs from total inhibition to normal growth filling the whole Petri dish can be visualized in Figure 4.

### 2.3. Response of Penicillium italicum towards 30 EOs

**Figure 5:**
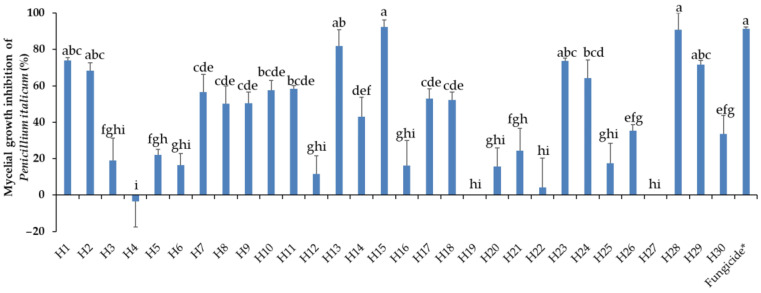
Mycelial growth inhibition of *P. italicum* by the thirty essential oils used at 0.5 mg/mL. H1, *C. martinii*; H2, *F. vulgare*; H3, *L. nobilis*; H4, *M. alternifolia*; H5, *S. sclarea*; H6, *C. odoranta*; H7, *P. cablin*; H8, *M. quinquenervia*; H9, *B. carterii*; H10, *L. angustifolia*; H11, *C. sinensis*; H12, *C. paradisii*; H13, *S. aromaticum*; H14, *M. piperita*; H15, *G. fragrantissima*; H16, *A. dracunculus*; H17, *C. limon*; H18, *D. carota*; H19, *Z. officinale*; H20, *P. crispum*; H21, *C. reticulata*; H22, *C. aurantium bergamia*; H23, *C. nardus*; H24, *P. asperum*; H25, *J. communis*; H26, *C. nobile*; H27, C. atlantica; H28, C. verrum; H29, C. sempervirens; H30, *O. basilicum*, after 23 days of incubation at 10 °C in the dark. * 25 g/L difenoconazole + 25 g/L fludioxonil. Data are expressed as means ± SE (n = 3). Data with different letters are significantly different (*p* ≤ 0.05, Fisher’s LSD tests).

**Figure 6:**
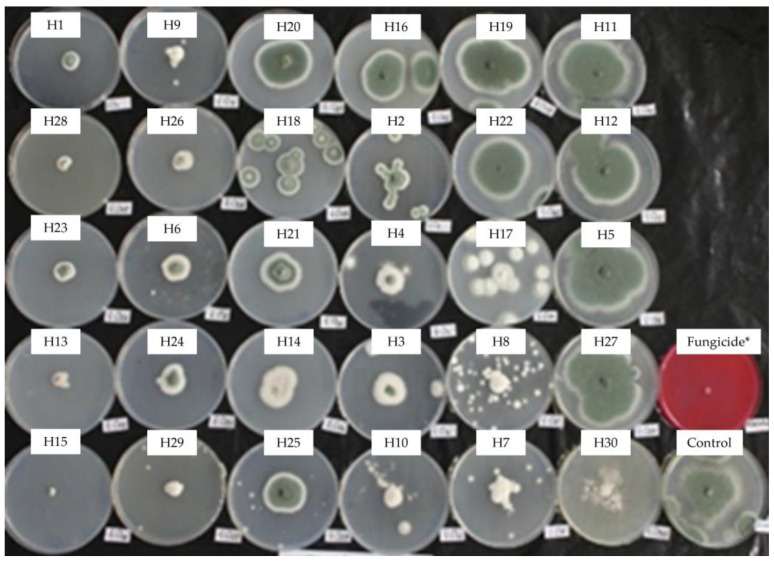
Mycelial growth inhibition of *P. italicum*i in PDA amended with the essential oils at 0.5 mg/mL. H1, *C. martinii*; H2, *F. vulgare*; H3, *L. nobilis*; H4, *M. alternifolia*; H5, *S. sclarea*; H6, *C. odoranta*; H7, *P. cablin*; H8, *M. quinquenervia*; H9, *B. carterii*; H10, *L. angustifolia*; H11, *C. sinensis*; H12, *C. paradisii*; H13, *S. aromaticum*; H14, *M. piperita*; H15, *G. fragrantissima*; H16, *A. dracunculus*; H17, *C. limon*; H18, *D. carota*; H19, *Z. officinale*; H20, *P. crispum*; H21, *C. reticulata*; H22, *C. aurantium bergamia*; H23, *C. nardus*; H24, *P. asperum*; H25, *J. communis*; H26, *C. nobile*; H27, C. atlantica; H28, C. verrum; H29, C. sempervirens; H30, *O. basilicum*, after 23 days of incubation at 10 °C in the dark. * 25 g/L difenoconazole + 25 g/L fludioxonil.

Mycelial growth of *P. italicum* in PDA was assessed 23 days post incubation at 10 °C in the dark. The activity of the EOs towards *P. italicum* varied greatly from lack of efficiency to highly effective, as expressed in Figure 5, showing a total lack of efficiency from −1.9% (H4) to 98% (H15). No significant differences were registered between the fungicide and the EOs H1, H2, H13, H15, H23, H28, and H29. Intermediate reactions were expressed by different EOs. Mycelial growth inhibition by respective EOs can be visualized in Figure 6.

### 2.4. Comparison between the Essential Oil Inhibitory Activity and between Fungi Tolerance

**Figure 7:**
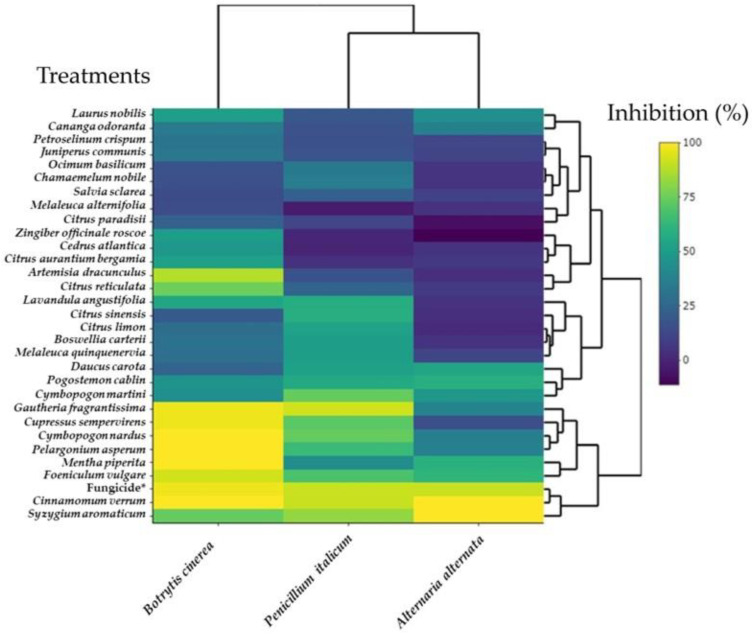
Heat map of the mycelial growth inhibition (%) of *B. cinerea*, *P. italicum*, and *A. alternata* by thirty essential oils at 8–23 days post-inoculation. Positive control is fungicide *: 25 g/L difenoconazole + 25 g/L fludioxonil.

The antifungal activity heat map of 30 EOs toward *B. cinerea*, *P. italicum*, and *A. alternata* is summarized in Figure 7. The heat map showed valuable information concerning the interaction between fungal species and the EOs. There are two clusters for the fungal species, one for *B. cinerea* and another for *A. alternanta* and *P. italicum*, having a similar mycelial growth inhibition pattern. The EOs of *G. fragrantissima* (H15), C. sempervirens (H29), *C. nardus* (H23), *P. asperum* (H24), *M. piperita* (H14), *F. vulgare* (H2), C. verrum (H28), and *S. aromaticum* (H13) were grouped in a separate cluster exerting a higher inhibition to *B. cinerea* compared to *A. alternata* and *P. italicum* that were less sensitive to the same EOs. This cluster also includes the fungicide. On the other hand, the EOs of *C. paradisii* (H12), *M. alternifolia* (H4), *S. sclarea* (H5), *C. nobile* (H26), *O. basilicum* (H30), *J. communis* (H25), *P. crispum* (H20), *C. odoranta* (H6), and *L. nobilis* (H3) were distinct in another cluster characterized by a level of inhibition of the three fungi lower than 50%.

### 2.5. Minimum Inhibitory Concentration

Different EO concentrations (0, 0.05, 0.1, 0.2, 0.3, and 0.4 mg/mL) were tested on six EOs selected based on two arbitrary criteria: (i) average mycelial growth inhibition of at least 78% towards the 30 EOs at 0.5 mg/mL, and (ii) inhibition of mycelial growth of at least 98% towards at least one out of the three fungal species. According to these criteria, the selected six EOs were Foeniculum vulgare (H2), Syzygium aromaticum (H13), Gautheria fragrantissima (H15), Cymbopogon nardus (H23), Pelargonium asperum (H24), and Cinnamomum verrum (H28) that were used to determine by linear regression the lowest concentration (MIC analysis) that exerted a total mycelial growth inhibition of each fungal species.

Linear regression was determined by relating mycelial growth inhibitions compared to the control to the corresponding concentration (0.05, 0.1, 0.2, 0.3, and 0.4 mg/mL) for every EO toward *A. alternata* (Table 1), *B. cinerea* (Table 2), and *P. italicum* (Table 3). Linear regression was performed for each pathogen. For *P. italicum*, linear regression was done only with the most effective EOs, which were Cinnamomum verrum (H28) and Syzygium aromaticum (H13).

**Figure 8:**
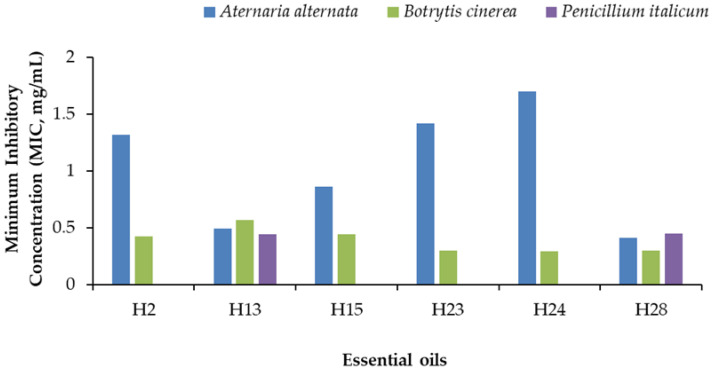
Minimum Inhibitory Concentration (MIC) of six essential oils against *A. alternata*, *B. cinerea*, and *P. italicum*. H2, *F. vulgare*; H13, *S. aromaticum*; H15, *G. fragrantissima*; H23, *C. nardus*; H24, *P. asperum*; H28, C. verrum.

Table 4 summarizes the equations of linear regression of every EO by fungal species. The coefficient of determination R^2^ of each equation was, in most cases, high, ranging between 0.96 and 0.62, except in three cases that were 0.27, 0.43, and 0.48. It is possible to determine the MIC using these equations by assigning the value 100 to y (y = 100) in order to find the value of x, which is the MIC. The results of the analysis are shown in Figure 8.

The EOs of H28 and H13 showed a broad spectrum of antifungal action since they completely inhibited mycelial growth of *A. alternata*, *B. cinerea*, and *P. italicum* at low concentrations (MIC) ranging between 0.31 and 0.45 mg/mL for H28 and between 0.37 and 0.57 mg/mL for H13. The low MIC values of these six EOs were registered against *B. cinerea*, ranging between 0.29 and 0.44 mg/mL. In comparison, the highest MIC values were towards *A. alternata* (0.85–1.71 mg/mL).

## 3. Discussion

In vitro analysis is the first step in a program aimed at studying a wide range of biological resources in order to identify the most bioactive products and to determine their working concentration range. This is particularly important for EOs since low concentrations can be ineffective, and high concentrations may result in phytotoxic or leave residues on treated plants [23]. Mani-Lopez et al. [24] pointed out that the antifungal activity of EOs must be demonstrated before undertaking analyses on target sites or mechanisms of action. This is true because, as far as we know, the activity of EOs varied greatly from highly effective to ineffective and from a general to a specific efficacy according to the target pathogen.

The use of 0.5 mg/mL was an appropriate dose to perform the right classification of the 30 EOs; such results were subsequently specified by MIC analyses. Our results were consistent concerning the effectiveness of cinnamon bark EO, which was a powerful product in reducing fungal growth at low doses (0.31–0.45 mg/mL) irrespective of the fungal species. Similarly, clove buds EO has also proven to be effective (0.37–0.57 mg/mL), whatever the fungal species, with an advantage of 0.06–0.11 mg/mL in favor of cinnamon bark EO. These two EOs are of particular interest for further studies on their use as a biological disinfectant, e.g., as a biological ingredient in a formulation suitable for post-harvest fruit coating.

In vitro studies by Sukatta et al. [25] using inverted Petri plates showed that the antifungal activity of clove and cinnamon volatile oils against six fungi caused the postharvest decay of grapes. An inverted Petri plate means the mycelium of the fungus is placed on the center of the Petri dish containing a nutrient medium such as PDA, and the oil pipetted on a filter paper is kept on the coverlid of the same Petri dish. They reported the MICs for the six fungi ranged from 200 to 800 mg mL^−1^ using clove bud EO and 50 to 800 mg mL^−1^ using cinnamon bark EO. This is in accordance with our results that cinnamon oil is slightly more effective against fungi than clove oil. Parker et al. [26] tested the antifungal activities of 21 EOs against human fungal pathogens such as *Candida auris*. They found the most effective EOs able to inhibit and kill the fungi were those of lemongrass, clove bud, and cinnamon bark when in direct contact and at concentrations safe for topical use.

In terms of chemical composition and according to the supplier of these EOs, E-cinnamaldehyde and cinnamyl acetate for cinnamon bark, eugenol and eugenyl acetate for clove buds are the major components of the respective EOs. This suggests that these oil constituents (E-cinnamaldehyde and cinnamyl acetate for cinnamon bark, eugenol and eugenyl acetate for clove buds) are owed to the fair effectiveness against the fungi tested in this research. The antifungal activities of cinnamon leaf EO were studied in vitro against wood-decay fungi in Taiwan. This study showed that this EO and its main component, cinnamaldehyde, exhibited strong antifungal activity at 50–100 ppm [27]. Our results and those of published works [28,29,30,31] agree that EOs from cinnamon bark or from clove bud possess a broad-spectrum antifungal activity.

Although from different plant species and harvested from different countries, the EOs of the aerial part of Ceylon lemongrass (H23) and that of geranium leaves (H24) unexpectedly showed similar patterns among the four fungal species. Thus, mycelial growth inhibition at 500 ppm was (99, 100, 81, 39%) and (100, 100, 71, 39%), respectively, for the four fungal species treated either with Ceylon lemongrass or with geranium leaves EOs (Table 2). Close chemical components of their respective EOs (citronellol and geraniol as major constituents) could explain this similarity of effectiveness characterized by a high activity against *B. cinerea*, a moderate activity towards *P. italicum*, but a low activity against *A. alternata*.

Methyl salicylate, a volatile form of salicylic acid responsible for increased membrane permeability, is the result of salicylic acid’s methylation. It can play an important role in systemic acquired resistance signaling and defense against pests, microbial pathogens, and antagonists [32,33]. Fragrant wintergreen leaves EO (H15), whose methyl salicylate is the major component, exhibited in our experiments high activity against the fungal species except for *A. alternata*, to which it was relatively tolerant at the concentration used. In another study, the fruit essential oil of *Alchornea cordifolia*, rich in methyl salicylate and citronellol, showed antifungal activity against *Aspergillus niger* and antibacterial activity against *Staphylococcus aureus* [34]. In addition to its antifungal activity, methyl salicylate was reported to increase the health-promoting properties of cherry fruit consumption with a positive effect on delaying the fruit postharvest senescence process [35]. Therefore, the EO of fragrant wintergreen leaves can play a double role in postharvest foodstuffs as an antifungal and an anti-senescence.

The essential oil of grapefruit peel (H12), whose main compound is limonene, showed the lowest efficacy against the four fungal species, in particular against *A. alternata* (−7%) and *P. italicum* (16%). In addition, the essential oils containing limonene, linalool, linalyl acetate, terpineol, or terpinene were the least effective, especially against *A. alternata* (1% to 14%) and to a lesser extent against *P. italicum* (−2% to 59%) or *B. cinerea* (22% to 83%). The EOs rich in limonene are from tea tree leaves (H4), the flowering top of clary sage (H5), sweet orange zest (H11), lemon zest (H17), fruit of black pepper (H20), mandarin zest (H21), and bergamot zest (H22). Thus, it seems that limonene has limited antifungal activity contrary to what has been observed with E-cinnamaldehyde or eugenol. However, Hao et al. [36] suggested that D-limonene has the potential to prevent the foodborne opportunistic yeast *Candida tropicalis* contamination in the food industry based on the observed disruption and alteration of cell wall integrity. Nevertheless, Kishore et al. [28] tested the effectiveness of five EO compounds (citral, eugenol, geraniol, limonene, and linalool) together with clove and cinnamon EOs for growth inhibition of 14 fungi using paper disc agar diffusion. They pointed out that limonene and linalool showed the least antifungal activity, which led to the removal of them from further tests. Such a result is in accordance with our finding that limonene and EOs rich in limonene (mainly those from citrus) are not advisable to consider as appropriate antifungal control agents because of their narrow spectrum antifungal activity.

Furthermore, our results allowed the issuing of a global classification concerning the degree of sensitivity of the fungal species by taking into account their reaction toward the totality of the EOs. *B. cinerea* was the most sensitive fungal species among the tested and, therefore, easier to control, especially when it is on apparent tissues such as those of post-harvest fruits. This pathogen is a serious problem for stored foodstuffs, even in cold storage. Several essential oils have shown high efficacy against it, which indicates the relative ease with which this pathogen could be controlled. *Cinnamomum zeylanicum* EO in the vapor phase was tested in vitro against *B. cinerea*, *Monilinia fructicola*, *Monilinia fructigena*, and *Monilinia laxa*. Based on mycelial growth inhibition, the volatiles of *C. zeylanicum* EO consistently showed higher inhibition against *B. cinerea*, suggesting a specific mode of action [37]. Vapor phase and direct contact via growth media of EOs could act differently against postharvest fungi [37,38], but this hypothesis needs confirmation. On the other hand, *A. alternata* and, to a lesser extent, *P. italicum* appear to have a greater ability to tolerate the various antifungal treatments. This property should be taken into account when a control strategy is planned.

Because of the tolerance observed in *Alternaria* spp. populations to different fungicides (QoIs, benzimidazole, fludioxonil, cyprodinil, boscalid, and pyraclostrobin) [39,40,41], it was suggested the need to study *Alternaria* isolates not previously exposed to fungicides to have a better estimate of the shifts in sensitivity [42]. Here, we have used different sources of EOs not previously applied as fungicides on *A. alternata*, and yet this tolerance was well observed. This tolerance could be due to an innate mechanism in this species (rigid cell wall, for example) allowing protection against adverse conditions. Furthermore, unlike some other fungal species, *Alternaria* spp. appear to be able to grow on host plants despite an environment characterized by water or thermal stress. Thus, Fagodiya et al. [42] emphasized that *Alternaria* blight severity in soybean fields increased with increasing temperature (19.8–32.5 °C) and sunshine hours, decreasing rainfall and relative humidity. Any agronomic factor that increases stress on the crop can act as a precursor for *Alternaria* infection, as well as the severity of the outbreak. Thus, there is evidence that *Aternaria* spp. possesses factors that allow it to withstand adverse conditions, including antifungal treatments, even before induction of mutation in response to any stress.

*Penicillium* spp. has also been relatively tolerant to many EOs. It appears that it uses a different mechanism than *Alternaria*, allowing it a moderate ability to tolerate an adverse environment such as antifungal compounds. Oiki et al. [43] tested *Penicillium* strains with no history of fungicide exposure, collected from various environmental and clinical sources, and despite this, they found fungicide-resistant strains, questioning where and how these *Penicillium* strains became resistant. The same situation arises in our analyses, suggesting that innate genetic variability in fungal species has occurred naturally without being related to a response to synthetic fungicide pressure.

## 4. Materials and Methods

### 4.1. Origin of the Essential Oils

Thirty EOs were offered for sale by a local company named STDCE (Société Tunisienne de Distribution et de Commerce Electronique, sarl) located at Megrine in the vicinity of Tunis, Tunisia. These EOs are packed in a dark glass bottle of 10 mL, which is marked with the logo ‘Aroma Vegetal’. Some oils were chosen without any prior information on their efficacy, while others were selected based on our recent results [2,44]. The chosen EOs are listed in Table 5, together with information given by the supplier about plant species, vegetal part of oil extraction, major chemical components, and the provenience of plant products.

### 4.2. Fungal Species

The fungal species *A. alternata*, *B. cinerea*, and *P. italicum* used in the present study were isolated from infected fruits (strawberry, peach, apricot, plum, nectarine, and orange) collected in cold storage. Pure cultures were transplanted into Petri dishes (diameter, 90 mm) with potato dextrose agar (PDA), then morphologically identified in the laboratory and conserved in PDA at 10 °C until use. These fungal species are considered the most damaging agents causing fruit decay during storage in Tunisia.

### 4.3. In Vitro Antifungal Activities on Mycelial Growth

The antifungal activities of EOs (Table 1) were assessed according to their contact phase effects on the mycelial growth of *A. alternata*, *B. cinerea*, and *P. italicum*. The EOs were dissolved in sterilized distilled water with 0.1% (*v*/*v*) Tween 20 (Sigma Aldrich, Steinheim, Germany) to obtain homogeneous emulsions, according to Moumni et al. [2,44]. The negative control was PDA containing 0.1% (*v*/*v*) Tween 20. The positive control was based on the fungicides as 25 g/L difenoconazole plus 25 g/L fludioxonil (Celest Extra 50 FS (CE); Syngenta, Cambridge, UK). The PDA was mixed and poured immediately into Petri dishes (diameter, 90 mm; 20 mL/plate), and after medium solidification, each plate was inoculated under aseptic conditions with a 6 mm plug, taken from the edge of actively growing cultures from each of the fungal species. The inoculated plates were sealed with Parafilm and incubated at 10 ± 2 °C in the dark for 7 to 23 days, depending on the speed of mycelial growth in the negative control at 10 °C of the fungal species. The orthogonal diameters of the colonies were measured when the negative control plates were completely covered by the mycelia.

A first experiment aimed to reveal the most effective EOs against each of the three fungal species was performed at a concentration of 0.5 mg/mL using the 30 EOs and the fungicide. After analysis, the retained EOs were used in a second experience by applying the EOs concentrations 0 (control, PDA), 0.05, 0.1, 0.2, 0.3, and 0.4 mg/mL in order to determine, by linear regression, the minimum concentration allowing 100% mycelial growth inhibition (MIC, minimum inhibitory concentration). The method used to dissolve the oils is the same as described above.

The experiments were carried out as three replicates per fungal species and treatment. Mycelial growth inhibition was calculated based on the Equation (1) [45]:

Mycelial growth inhibition (%) = [1 − dt/dc] × 100(1)

where dc and dt represent the mean diameter of the mycelial growth of the control and treated fungal species, respectively. The MIC for each selected EO and fungal species was determined based on the linear regression between EO concentrations (x) and respective mycelial growth inhibition (y = ax + b), considering (y) is equal to zero and the (x) is to be found out by this equation, which is the value of the MIC (mg/mL).

### 4.4. Statistical Analysis

For the first set of experiments using 30 EOs, each experimental unit was represented by one EO (concentration 0.5 mg/mL) and one fungal species repeated 3 times, knowing that the protocol was completely randomized and was composed of 30 EOs and 3 fungal species. For the second set of experiments concerning the MIC, also completely randomized, each experimental unit was constituted by one EO, one fungal species, and one EO concentration. The protocol included 6 EOs at 6 concentrations each and 3 fungal species. Excel (version 2016) was used to determine the average, standard errors, and linear regression. All of the trials were repeated at least twice, and data are means ± standard error (SE) presented as histograms or linear regression equations. Analysis of variance was calculated using SPSS (version 20; IBM, Armonk, NY, USA). The data for inhibition of mycelial growth underwent analysis of variance (ANOVA). Means were compared using Fisher’s tests for protected least significant difference (LSD) at *p* < 0.05. Package ‘heatmaply’ was used to prepare the heat map figure with RStudio version 2023.03.0+386.

## 5. Conclusions

The present study has demonstrated the in vitro activities of thirty EOs and their effectiveness against the three important fungal pathogens: *A. alternata*, *B. cinerea*, and *P. italicum*. *C. verrum* and *S. aromaticum* EOs at 0.5 mg/mL presented the highest inhibitory activity for the three pathogens. These two EOs are promising biological candidates to be included in our next program as essential preservative ingredients in the coating formulation for postharvest fruits during storage to maintain the quality and extend shelf life.

## Figures and Tables

**Table 1:** Mycelial growth inhibition of *A. alternata* by the six essential oils from Foeniculum vulgare (H2), Syzygium aromaticum (H13), Gautheria fragrantissima (H15), Cymbopogon nardus (H23), Pelargonium asperum (H24), and Cinnamomum verrum (H28) after 21 days of incubation at 10 °C in the dark.

Essential Oil	Inhibition of Mycelial Growth of *A. alternata* (%) at Different Essential Oil Concentrations (mg/mL)				
	0.05	0.1	0.2	0.3	0.4
*Foeniculum vulgare* (H2)	3.8 ± 0.9 bc	6.5 ± 1.5 bc	9.4 ± 5.08 c	17.6 ± 6.1 cd	28.2 ± 8.8 b
*Syzygium aromaticum* (H13)	18.7 ± 8.5 a	17.8 ± 5.3 ab	65.0 ± 2.8 a	76.7 ± 4.4 b	86.9 ± 6.7 a
*Gautheria fragrantissima* (H15)	7.2 ± 5.2 ab	2.9 ± 1.1 c	27.5 ± 9.7 b	26.4 ± 13.8 c	28.4 ± 11.2 b
*Cymbopogon nardus* (H23)	−6 ± 0.3 c	−1.3 ± 3.7 c	3.8 ± 1.48 d c	5.8 ± 3.9 d	21.4 ± 5.1 b
*Pelargonium asperum* (H24)	1.3 ± 0.7 bc	2.0 ± 1.7 c	7.4 ± 2.7 c	0.0 ± 0.6 d	23.9 ± 21.4 b
*Cinnamomum verrum* (H28)	10.3 ± 2.6 ab	26.7 ± 13.5 a	53.1 ± 7.4 a	99.4 ± 0.4 a	100.00 a

Data are expressed as means ± SE (n = 3). Data with different letters in the same column are significantly different (*p* ≤ 0.05, Fisher’s LSD tests).

**Table 2:** Mycelial growth inhibition of *Botrytis cinerea* by the six essential oils from *Foeniculum vulgare* (H2), *Syzygium aromaticum* (H13), *Gautheria fragrantissima* (H15), *Cymbopogon nardus* (H23), *Pelargonium asperum* (H24), and *Cinnamomum verrum* (H28) after 21 days of incubation at 10 °C in the dark.

Essential Oil	Inhibition of Mycelial Growth of *B. cinerea* (%) at Increasing Essential Oil Concentrations (mg/mL)				
	0.05	0.1	0.2	0.3	0.4
*Foeniculum vulgare* (H2)	0 ± 0 c	31.9 ± 8.8 cd	89.8 ± 0.7 ab	84.4 ± 7.7 ab	94.1 ± 1.5 ab
*Syzygium aromaticum* (H13)	46.6 ± 7.7 a	49.6 ± 1.3 bc	63.1 ± 2.0 c	73.5 ± 1.6 bc	72.4 ± 0.5 c
*Gautheria fragrantissima* (H15)	26.2 ± 24.9 abc	58.1 ± 14.5 b	88.7 ± 5.6 ab	77.4 ± 2.7 bc	90 ± 4.8 b
*Cymbopogon nardus* (H23)	42.9 ± 4.2 ab	85 ± 3.1 a	89.8 ± 1.9 ab	90.4 ± 0.9 ab	99.1 ± 0.2 a
*Pelargonium asperum* (H24)	12.3 ± 12.0 bc	13.6 ± 5.4 d	25.6 ± 6.3 d	63.1 ± 14.5 c	65.5 ± 3.6 c
*Cinnamomum verrum* (H28)	12.3 ± 2.6 bc	25.7 ± 0.7 d	96.3 ± 2.1 a	99.8 ± 0.1 a	100 a

Data are expressed as means ± SE (n = 3). Data with different letters in the same column are significantly different (*p* ≤ 0.05, Fisher’s LSD tests).

**Table 3:** Mycelial growth inhibition of Penicillium italicum by the two essential oils of Syzygium aromaticum (H13) and Cinnamomum verrum (H28) after 23 days of incubation at 10 °C in the dark.

Essential Oil	Inhibition of Mycelial Growth of *P. italicum* (%) at Increasing Essential Oil Concentrations (mg/mL)				
	0.05	0.1	0.2	0.3	0.4
*Syzygium aromaticum* (H13)	49.9 ± 15.5 a	55.1 ± 14.0 a	76.8 ± 3.3 a	87.8 ± 2.3 a	87.4 ± 3.3 b
*Cinnamomum verrum* (H28)	14.0 ± 2.6 a	10.4 ± 5.1 b	30.3 ± 19.5 a	76.1 ± 12.9 a	100 a

Data are expressed as means ± SD (n = 3). Data with different letters in the same column are significantly different (*p* ≤ 0.05, Fisher’s LSD tests).

**Table 4:** The equation of linear regression and the corresponding determination coefficient R^2^ determined for every fungal species and essential oil.

Fungal Species	Code of EOs	Linear Regression	R²
*Alternaria alternata*	H2	y = 0.0748x + 1.5905	0.96
	H13	y = 0.1849x + 9.2307	0.88
	H15	y = 0.1199x − 3.0083	0.82
	H23	y = 0.0737x − 7.8006	0.85
	H24	y = 0.0607x − 3.8176	0.74
	H28	y = 0.2279x + 5.4806	0.90
*Botrytis cinerea*	H2	y = 0.2143x + 9.6257	0.79
	H13	y = 0.1268x + 27.743	0.72
	H15	y = 0.1751x + 22.99	0.76
	H23	y = 0.1585x + 37.839	0.62
	H24	y = 0.1133x + 66.675	0.27
	H28	y = 0.2816x + 12.828	0.77
*Penicillium italicum*	H13	y = 0.1589x + 29.716	0.77
	H28	y = 0.2258x − 2.5463	0.94

**Table 5:** Details of the thirty essential oils used in this study, including the main volatile constituents defined according to the supplier.

Code	Plant Species	Vegetal Part of Oil Extraction	Major Chemical Components	Origin of Plant Product
H1	*Cymbopogon martinii*	Aerial part of palmarosa	Geraniol, geranyl acetate	India
H2	*Foeniculum vulgare*	Aerial part of sweet fennel	Trans-anethol, limonene	France
H3	*Laurus nobilis*	Leaves of noble laurel	1,8-cineole, terpenyl acetate	Balkans
H4	*Melaleuca alternifolia*	Leaves of tea tree	Terpineol, alpha-terpinen	Australia
H5	*Salvia sclarea*	flowering top of clary sage	Linalyl acetate, linalol	Bulgaria
H6	*Cananga odoranta*	Flower of ylang ylang	Germacrene D, benzyle acetate and banzaoate, farnesene	Comores
H7	*Pogostemon cablin*	Flowering top of patchouli	Patchoulol, alpha-patchoulene, alpha-bulnesene	Indonesia
H8	*Melaleuca quinquenervia*	Leaves of niaouli	1,8 cineole, α-pinene, viridiflorol	Madagascar
H9	*Boswellia carterii*	Oleoresin	α-Pinene; β-Myrcene; β-Caryophyllene	Somalia
H10	*Lavandula angustifolia*	Flowering top of fine lavender	Linalyle acetate, linalol, camphor	New Zealand
H11	*Citrus sinensis*	Sweet orange zest	Limonene	Greece
H12	*Citrus paradisii*	Grapefruit zest	Limonene	Argentina
H13	*Syzygium aromaticum*	floral buds of cloves	Eugenol, eugenyl acetate	Madagascar
H14	*Mentha piperita*	Aerial part of pepper mint	Menthol, menthone	France
H15	*Gautheria fragrantissima*	Fragrant wintergreen leaves	Methyl aalicylate	Nepal
H16	*Artemisia dracunculus*	Flowering top of tarragon	Estragole, chavicol	France
H17	*Citrus limon*	Lemon zest	Limonene, limonum citrus	Italy
H18	*Daucus carota*	Seeds of carrot	Carotol, alphapinene	France
H19	*Zingiber officinale*	Rhizome ofginger	zingeberene, gingerol, bisabolene	China
H20	*Petroselinum crispum* (*Piper nigrum*)	Fruit of black pepper	Caryophyllene, limonene	Madagascar
H21	*Citrus reticulata*	Mandarin zest	Limonene, γ-Terpinene	Argentina
H22	*Citrus aurantium bergamia*	Bergamot zest	Limonene, linalyl acetate, linalol	Italy
H23	*Cymbopogon nardus*	Aerial part of Ceylon lemongrass	Citronellal, geraniol, citronellol, limonene, elemol	Ceylon
H24	*Pelargonium asperum*	Geranium leaves	Citronellol, geraniol	Egypt
H25	*Juniperus communis*	Branch of genevrier	α-Pinene, Sabinene	Bulgaria
H26	*Chamaemelum nobile*	Flowers of chamomile	Isobutyl angelate, Isoamyl angelate, methylallyl angelate	Italy
H27	*Cedrus atlantica*	Wood of Atlas cedar	Himachalene	Morocco
H28	*Cinnamomum verrum*	Cinnamon bark	E-cinnamaldehyde, cinnamyl acetate	Madagascar
H29	*Cupressus sempervirens*	Branch of cypress	Alpha pinene, Delta 3 carene	Morocco
H30	*Ocimum basilicum*	Flowering top of basil	Estragol (Methylchavicol)	Italy

## Data Availability

Data are contained within the article.
